# Electroencephalography and mild cognitive impairment research: A scoping review and bibliometric analysis (ScoRBA)

**DOI:** 10.3934/Neuroscience.2023012

**Published:** 2023-06-13

**Authors:** Adi Wijaya, Noor Akhmad Setiawan, Asma Hayati Ahmad, Rahimah Zakaria, Zahiruddin Othman

**Affiliations:** 1 Department of Health Information Management, Universitas Indonesia Maju, Jakarta, Indonesia; 2 Department of Electrical and Information Engineering, Universitas Gadjah Mada, Yogyakarta, Indonesia; 3 School of Medical Sciences, Health Campus, Universiti Sains Malaysia, 16150 Kota Bharu, Malaysia

**Keywords:** electroencephalography, mild cognitive impairment, bibliometric analysis, scoping review, ScoRBA

## Abstract

Mild cognitive impairment (MCI) is often considered a precursor to Alzheimer's disease (AD) and early diagnosis may help improve treatment effectiveness. To identify accurate MCI biomarkers, researchers have utilized various neuroscience techniques, with electroencephalography (EEG) being a popular choice due to its low cost and better temporal resolution. In this scoping review, we analyzed 2310 peer-reviewed articles on EEG and MCI between 2012 and 2022 to track the research progress in this field. Our data analysis involved co-occurrence analysis using VOSviewer and a Patterns, Advances, Gaps, Evidence of Practice, and Research Recommendations (PAGER) framework. We found that event-related potentials (ERP), EEG, epilepsy, quantitative EEG (QEEG), and EEG-based machine learning were the primary research themes. The study showed that ERP/EEG, QEEG, and EEG-based machine learning frameworks provide high-accuracy detection of seizure and MCI. These findings identify the main research themes in EEG and MCI and suggest promising avenues for future research in this field.

## 1. Introduction

The most common type of neurodegenerative dementia is Alzheimer's disease (AD), with 10 million new dementia cases identified each year [1]. AD is the most common cause of dementia, accounting for 60–70% of these new cases, followed by vascular dementia (VaD), dementia with Lewy bodies (DLB), and other forms of neurodegenerative illnesses [1]. Before the development of dementia, individuals often experience two stages of decreased cognition—subjective cognitive decline (SCD), which is not corroborated by an informer or neuropsychological testing [2], and mild cognitive impairment (MCI), which is corroborated by an informer or neuropsychological testing [3]. In both stages, individuals can undertake personal and instrumental daily activities [2],[3]. According to the Alzheimer Association [4], 15% of MCI in individuals over 65 years of age progress to dementia; however, in a different study [5], 32% of individuals with MCI were found to have AD at the 5-year follow-up. Older age, poor cognition, APOE 4 allele carrier status, and hypertension could enhance dementia risk [6]. Thus, it is important to establish biomarkers that can identify those at high risk for dementia and, ideally, its cause.

Studies combining cognitive assessments with cerebrospinal fluid (CSF) proteins, magnetic resonance imaging (MRI), and fluorodeoxyglucose-positron emission tomography (FDG-PET) have shown outstanding results in detecting MCI patients who later develop dementia [7]–[9]. However, these biomarkers are time-consuming and expensive, and some are invasive, and, therefore, not suitable for daily clinical practice, except in subspecialized settings. Electroencephalography (EEG) is a potential method for evaluating variations in brain activity between MCI and AD patients from healthy cohorts [10].

EEG is a low-cost, non-invasive, and portable technique that directly monitors brain activity with a high temporal resolution. It has been used for diagnosing and managing epilepsy and other neurological disorders. Clinical evidence supporting the use of EEG can be found in studies on the assessment and diagnosis of cognitive impairment [11],[12], traumatic brain injury [13], prediction of tinnitus treatment [14], and diagnosis of neurodegenerative diseases and depression [15]. Several types of EEG are commonly employed in research and clinical settings, including resting-state EEG, event-related potential (ERP), quantitative EEG (QEEG), source localization EEG, high-density EEG, and intracranial EEG. Some of the most promising EEG biomarkers for the early detection of AD due to their strong correlations with cognitive function include decreased alpha and beta rhythm activity, increased delta and theta oscillations, diminished complexity and coherence in EEG recordings, and decreased ratios of theta/gamma and high alpha/low alpha [11],[16]. Poor spatial resolution [17], necessitating a large number of trials [18], and susceptibility to artifacts that can contaminate EEG signals [19] are some of the limitations of EEG.

In this study, we aimed to conduct a scoping review of EEG and MCI research aided by bibliometric analysis (ScoRBA) and have presented our findings using the Patterns, Advances, Gaps, Evidence of Practice, and Research Recommendations (PAGER) framework introduced by Bradbury-Jones et al. [20]. This approach will provide valuable insights into the current state of EEG and MCI research, identify gaps in knowledge, and provide recommendations for future research directions. By examining the existing literature, we aim to contribute to the advancement of EEG as a potential tool for the early detection and assessment of cognitive impairment, including MCI, in individuals.

## 2. Materials and methods

The methodology employed for the scoping review in this study was adapted from Arksey and O'Malley's approach [21]. The five key steps of Arksey and O'Malley's approach are summarized in Table 1. The first step, which was to identify the primary research question, was deliberated in the introductory section. On September 20, 2022, a literature search was conducted using the Scopus database (Step 2). Scopus was selected as the database due to its extensive coverage of journals and citations [22],[23].

**Table 1.** Five-step scoping review framework [21].

**Step**	**Description**
1. Identification of research questions and related studies	The research question in this paper is “What are the main themes in the EEG and MCI research?”
2. Identification of related studies	A preliminary reading of relevant studies was performed to determine the keywords and terms utilized in the article selection.
3. Study selection	Peer-reviewed original articles published in the Scopus-indexed journals written in English were selected. Articles that assessed the use of EEG in patients with MCI met the selection criteria for this study.
4. Charting the data	The co-occurrence keyword was used to group the data into a graphical representation (See section 3.2).
5. Collating, summarizing, and reporting the results	The Patterns, Advances, Gaps, Evidence of Practice, and Research Recommendations (PAGER) framework was used to summarize, analyze, and present the results in order to enhance the quality and applicability of the literature review (see section 4), as suggested by Bradbury-Jones et al. [20]

**Figure 1.**
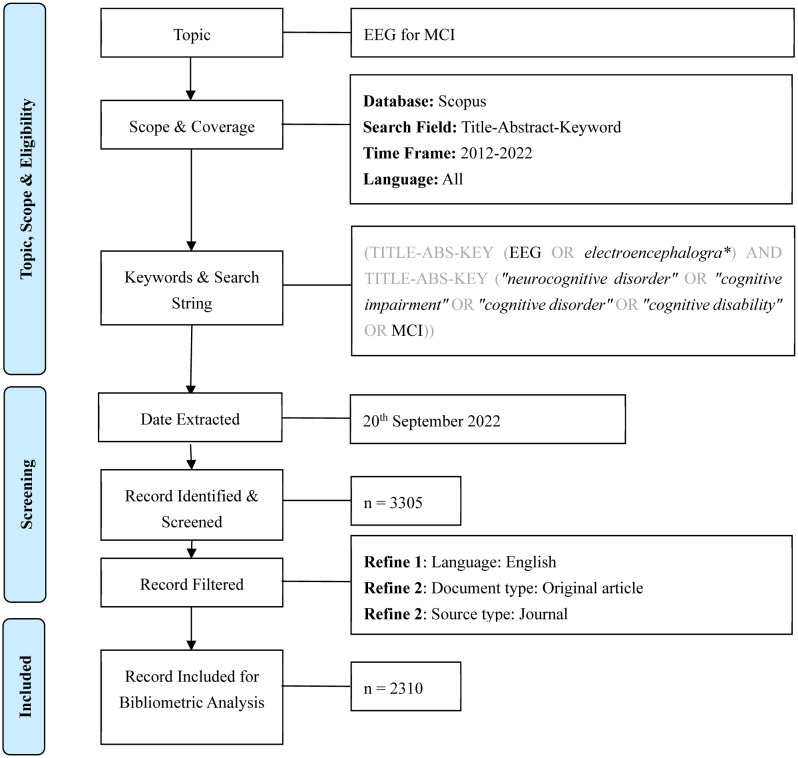
PRISMA flow diagram of the search strategy [24].

The search strategy employed the following search phrases: “TITLE-ABS-KEY (EEG OR electroencephalogra*)” AND TITLE-ABS-KEY (“neurocognitive disorder” or “cognitive impairment” or “cognitive disorder” or “cognitive disability” OR MCI). These search phrases were designed to capture the relevant documents related to EEG and the various terms associated with cognitive impairment or disorders. For the inclusion of documents in the review, specific criteria were applied. Only journal articles published in the English language between 2012 and 2022 were considered eligible for inclusion (Step 3). These criteria were established to focus on recent research and ensure that language barriers did not hinder the selection process. The selection of documents was based on a thorough examination of titles, abstracts, and keywords to determine their relevance to the scope of the study. The PRISMA flow diagram presented in Figure 1 was utilized to visually represent the search procedure and to provide transparency in the document selection process. This diagram outlines the number of documents identified during the initial search, the documents excluded after the screening, and the final number of documents included in the scoping review [24]. Steps 4 and 5 are explained in Sections 3.2 and 4.

## 3. Results

The initial search results from the Scopus database generated 3,305 documents published from 2012 until September 2022 (Figure 1). After removing papers in languages other than English, document types other than original articles, and source types other than journals, 2,310 documents remained. This step ensured that high-quality, peer-reviewed original articles (primary research documents) were included.

### 3.1. Publication output

**Figure 2.**
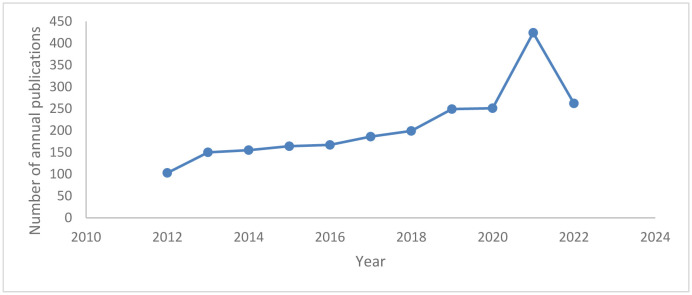
The number of annual publications on EEG and MCI research was published from 2012 to September 2022 in the Scopus database.

The number of articles showed an increasing trend from 2012 to 2021. There was a drop in the number of articles in 2022 due to incomplete data (Figure 2).

**Figure 3.**
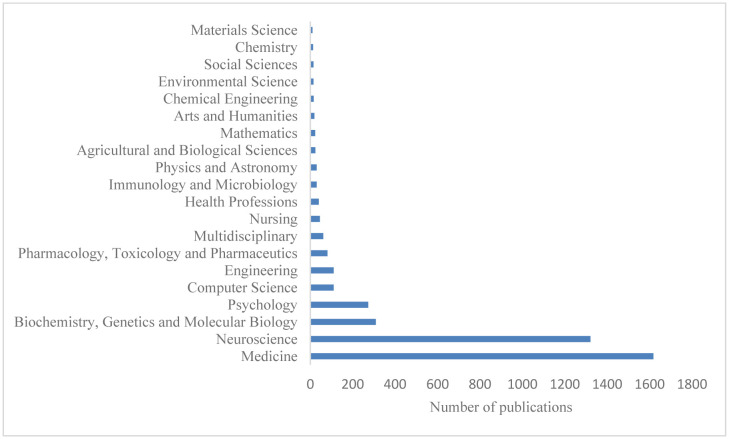
Top 20 subjects in the area of EEG and MCI research.

The research output on EEG and MCI research covered 24 different subject areas. Medicine, neuroscience, biochemistry, genetics and molecular biology, and psychology were the four subject areas that made up the most articles (3,521/4,172, or 84.40% of the total), followed by computer science, engineering, pharmacology, toxicology and pharmaceutics, health professions, multidisciplinary professions, etc. Figure 3 lists the top 20 EEG and MCI research areas currently under investigation.

### 3.2. Data Charting (Step 4)

We changed the method of data charting in our scoping review process from the narrative review approach to the use of co-occurrence keyword analysis, one of the bibliometric analysis techniques (ScoRBA). The narrative review strategy often entails focusing on major themes or concepts found in the literature by summarizing and synthesizing the results from the chosen documents in a narrative or descriptive way. However, co-occurrence keyword analysis, a quantitative approach, looks at patterns of word co-occurrence within the chosen documents. Researchers can find hidden patterns, correlations, and themes in the literature by using co-occurrence keyword analysis, which might not have been obvious through a typical narrative.

**Figure 4.**
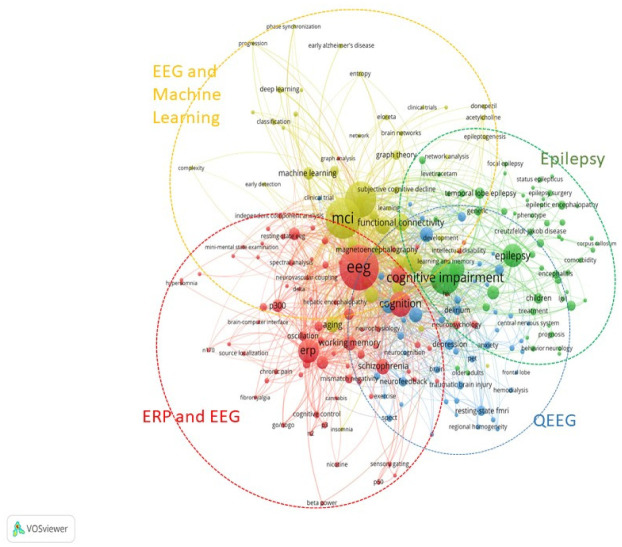
Co-occurrence of author keywords. 229 out of 5002 keywords met the threshold of a minimum of five occurrences.

In our study, a co-occurrence keyword analysis was conducted to gain insights into the main themes and clusters within EEG and MCI research. All 2,310 documents were downloaded from the Scopus database in the Comma-Separated Values (.csv) format and analyzed using VOSviewer version 1.6.17 [25] for co-occurrence keyword analysis. A total of 5,002 author keywords were initially selected from our collection of 2,310 documents. To ensure robust analysis, we focused on keywords that appeared at least five times, resulting in the selection of 229 keywords out of the initial pool. Our keyword analysis revealed the presence of distinct clusters within the keyword network, as illustrated in Figure 4. Based on the dominant keywords within each cluster, we categorized the research into the following clusters and themes (Table 2). The key clusters and themes were then used to highlight important patterns in the PAGER framework.

**Table 2.** Four clusters and themes within EEG and MCI research.

**Cluster**	**Theme**	**Description**
1. Red	ERP and EEG	The red cluster, comprising 80 keywords, predominantly focused on event-related potential (ERP) and EEG. This theme suggests a significant body of research exploring the relationship between EEG patterns and cognitive processes in MCI patients.
2. Green	Epilepsy	The green cluster, consisting of 56 keywords, centered around epilepsy. This theme indicates that a substantial portion of the research on EEG and MCI pertains to the overlap between these two conditions, likely investigating the use of EEG in diagnosing or monitoring MCI patients with comorbid epilepsy.
3. Blue	Quantitative EEG (QEEG)	The blue cluster, comprising 47 keywords, emphasized QEEG. This theme suggests that the research in this cluster focuses on the quantitative analysis of EEG signals to derive meaningful insights regarding MCI and the related cognitive impairments.
4. Yellow	EEG and machine learning	The yellow cluster, encompassing 46 keywords, revolved around the intersection of EEG and machine learning. This theme indicates an emerging area of research exploring the application of machine learning techniques to EEG data to diagnose, predict, or monitor patients with MCI.

## 4. Discussion

AD pathology can be studied effectively through the use of EEG-based brain mapping [16]. By analyzing the brain's electrical activity, EEG permits researchers to investigate the neurophysiological alterations associated with AD [26]. EEG-based brain mapping is essential for advancing our understanding of AD pathology and facilitating the development of effective diagnostic and treatment tools for this debilitating disease. To improve the quality and applicability of our scoping review, the PAGER framework [20] was utilized in the final step (Step 5) of Arksey and O'Malley's approach [21]. Table 3 summarizes the key findings of the PAGER framework, including model themes, research advances, research gaps, evidence for practice, and research recommendations.

**Table 3.** Results of the PAGER analysis of the EEG and mild cognitive impairment research.

**Patterns**	**Advances**	**Gaps**	**Evidence for practice**	**Research recommendation**
ERP and EEG (Red)	Combined EEG/ERP measures of memory as a diagnostic tool for detecting MCI or prodromal AD [27].	There is a need to understand the causal effects between amyloid β depositions and neural excitability [28].	EEG/ERP as biomarkers for assessing early memory decline and treatment response [29].	Future studies should establish ERP's sensitivity and specificity in discriminating AD, MCI, and cognitively intact AD-risk groups as well as measuring late cognitive ERP during complicated tasks, especially in healthy older adults at risk for cognitive decline.
Epilepsy (Green)	Knowledge discovery from machine learning classifiers, such as seizure localization, which points to the affected brain lobe(s) and channel importance and based on participating channels in a seizure may help predict cognitive impairment secondary to seizures, EEG interictal spikes (IIS), and antiepileptic medicines [30],[31].	There is a need to select machine learning classifiers and features for high-accuracy seizure detection and to predict cognitive impairment [30],[31].	EEG is currently used to detect seizures [32] and cognitive impairment in epilepsy disorders [33]–[35].	Future studies should explore the associations between cognitive deficits and seizure timing (occurrence).
QEEG (Blue)	Development of simple diagnostic algorithms using spectral analysis of EEG to detect MCI cases [36].	There is a need to find clear-cut EEG signs and correlations with neuropathology and neuropsychology for consistent and precise assessments [37],[38].	QEEG is a diagnostic and prognostic biomarker and monitors treatment response in MCI cases [36],[39],[40].	Future large-scale longitudinal clinical studies are required to determine the diagnostic and prognostic potential of QEEG measurements as early functional markers of AD on a subject-by-subject basis.
EEG and machine learning (Yellow)	Machine learning for extracting the relevant EEG/ERP data for cognitive assessment [41],[42].	There is a need to select machine learning classifiers and features for a high-accuracy cognitive assessment [43].	EEG-based machine learning frameworks for accurate diagnosis of MCI cases [44]–[51].	Future studies to utilize a machine learning framework for extracting the most relevant features from EEG data empirically thereby resulting in high accuracy of cognitive assessment.

### 4.1. EEG/ERP and mild cognitive impairment

The first report on using scalp EEG was published in 1929 [52]. EEG is a cost-effective and non-invasive technology that directly assesses the mean electrical activity of the brain at scalp sites with excellent temporal precision (milliseconds) [53]. ERP is one of the most extensively used EEG techniques to examine brain activity in response to sensory, motor, or cognitive events. Several ERP components (e.g., N1, P2, and P3) can provide full information about low (basic sensory and perceptual processing) or high (attention, memory, learning, problem-solving, and communication) level cognitive activities of the brain [54] and have been used in research for several decades.

Three EEG measures that have been found to strongly correlate with verbal learning and memory abilities in healthy elderly people and patients with MCI or prodromal Alzheimer's disease (AD) in previous research include ERP P600 [27], suppression of oscillatory activity in the alpha frequency range [55]–[59], and cross-frequency coupling between low theta/high delta and alpha/beta activity. Xia and colleagues [29] evaluated the relationships among three previously identified electrophysiological measures: the P600 ERP and two oscillatory effects (i.e., alpha suppression and φ/δ-α/β coupling), as well as their potential as biomarkers of verbal memory functioning in healthy aging, MCI, and prodromal Alzheimer's disease. The study highlighted the importance of combining ERP and EEG oscillatory measures as predictors of verbal memory in individuals with MCI. Recent research by Meghdadi et al. [60] demonstrated that subtle resting-state EEG abnormalities can differentiate between the MCI and AD groups. The study concluded that the MCI group demonstrated a moderate increase in δ, φ, and Theta-to-Alpha (TAR) ratio, mostly in temporal channels [60].

A systematic literature review investigating ERP as a potential biomarker of AD-related neuropathology [61]. The patterns of cognitive ERP (≥150 ms post-stimulus) differentiate MCI, AD, and cognitively intact elders who are at risk for AD due to the Apolipoprotein-E ε4 allele (ε4+) from cognitively healthy older adults. The review also implies that the integration of ERP into cognitive assessment may significantly improve the early identification and characterization of brain dysfunction, allowing for more rapid differential diagnosis and intervention targeting [61]. Future research is necessary to establish ERP's sensitivity and specificity in discriminating AD, MCI, and cognitively intact AD-risk groups as well as measuring late cognitive ERP during complicated tasks, especially in healthy older adults at risk for cognitive decline.

In a more recent study, Devos et al. [28] discovered a correlation between neuronal hyperexcitability and elevated amyloid levels in cognitively healthy older individuals. Cognitively healthy older individuals with elevated amyloid levels process stimuli less efficiently than those without elevated amyloid levels, as indicated by the smaller P3 ERP task difference compared to those without elevated amyloid levels. Together, these findings and the absence of statistically significant differences in behavioral outcomes suggest that enhanced amyloid deposition-induced hyperexcitability is a non-functional neuronal compensation that may eventually impair working memory. To confirm this observation, it is necessary to comprehend the causal effects of amyloid deposition and neural excitability.

### 4.2. Epilepsy and mild cognitive impairment

Epileptic seizures are brief bursts of excessively synchronized brain activity [62]. They cover a wide range of occurrences, from mild clinical manifestations, such as brief and hardly detectable loss of consciousness, to vigorous episodes of muscular shaking that may cause physical harm [63]. Epilepsy is diagnosed based on EEG abnormalities, including seizures and interictal epileptiform discharges (IEDs). EEG findings help categorize seizure types and epilepsy disorders [32].

EEG analysis of patients with recurrent seizures showed “subclinical” aberrant electrical activity between seizures. IEDs are classified based on their EEG signatures—sharp waves, spikes, sharp waves/spikes-and-slow waves, and numerous spikes-and-slow waves [64]. However, background noise and aberrations, such as eye blinks and muscle movements, can contaminate EEG readings, causing electrical interference that is difficult to detect visually in longer recordings. Thus, there is a need to select machine learning classifiers and features for high-accuracy seizure detection and to predict cognitive impairment [31].

Although seizures are the most obvious clinical manifestation of epilepsies, people with epilepsy are at risk of other health problems at a higher incidence rate than would be expected [44]. Common co-morbidities in epilepsy include cognitive impairment, such as memory, attention, and processing problems; mental health problems, such as depression and anxiety, and somatic co-morbidities, such as sleep disorders and migraines [33].

Most cognitive impairment in epilepsy is due to its etiology. Trauma, hypoxia, ischemia, and mesial temporal sclerosis secondary to prolonged seizures are acquired illnesses that can cause epilepsy and cognitive impairment. Genetic disorders, including tuberous sclerosis, Fragile X, Rett, and Dravet syndromes, can also lead to epilepsy and cognitive impairment. In addition to static abnormalities induced by the underlying etiology, temporary cognitive impairment may be due to seizures, EEG interictal spikes (IIS), and antiepileptic medicines. In many people, cognitive impairment is due to a combination of causes [33].

The age of seizure onset is another factor associated with cognitive impairment. When compared to healthy peers, children with epilepsy are likelier to have severe cognitive impairment, primarily affecting language, semantic, motor, and visual functions [34]. According to the same study, children with idiopathic epilepsy who have more nocturnal seizures than diurnal seizures have both overt and subtle cognitive abnormalities [34]. Future studies are needed to explore the associations between cognitive deficits and seizure timing (occurrence).

### 4.3. Quantitative EEG (QEEG) and mild cognitive impairment

The diagnosis of cognitive impairment and neurodegenerative illnesses using EEG relies heavily on the EEG expert's visual assessment. The limitations that come with this have been supplemented by the QEEG method, which extracts parameters from continuous variable data analysis. Currently, studies on brain function assessment and early diagnosis of degenerative brain illnesses, such as AD and AD-MCI, are advancing [39]. For instance, QEEG measurements appear to differentiate between different forms of dementia and correlate favorably with surrogate markers of AD neuropathology, making them promising low-cost and noninvasive diagnostics of AD. The diagnostic and prognostic value of QEEG measurements as early functional markers of AD should be determined in future extensive longitudinal clinical studies on a subject-by-subject basis.

One technique for analyzing QEEG signals is using the power spectrum, and this approach is a well-established core component of the general method utilized in clinical research. The most frequent spectral shift so far is thought to be connected to the degree of cognitive deterioration brought on by the rise in the slow wave of the EEG [36],[40],[66]. Spectral analysis of EEG is used to develop simple diagnostic algorithms that can identify MCI. However, finding distinct EEG signals with strong neuropathology and neuropsychology correlations is necessary for reliable and accurate assessments.

In a broad frequency range, spectral, and EEG complexity alterations can be detected even in the early stages of AD. However, applying traditional EEG analysis techniques alongside QEEG may further increase the likelihood of making an early diagnosis of AD [37]. A recent study by Engedal et al. [66] concluded that applying QEEG using the statistical pattern recognition (SPR) method could predict conversion to dementia in patients with SCD and MCI. The authors also suggested adding this method to other routine diagnostic methods, such as cognitive tests and other biomarkers, to benefit from the discriminant power of the proposed method. A study by Jeong et al. [36] demonstrated that SCD participants had more frontal delta waves and less occipital alpha1 compared to control participants. SCD is one of AD's early clinical signs and is linked to nerve degeneration. Hamilton et al. [38] showed that the slowing of QEEG may occur before the onset of dementia in individuals with eventually identified MCI. In addition, another study by Han and Youn [40] used QEEG to monitor the treatment response in MCI patients. Future studies should utilize QEEG characteristics, neuroimaging, and/or machine learning to better collect and show neural network activation.

### 4.4. EEG-based machine learning and mild cognitive impairment

Machine learning is a set of algorithms that enables the automatic detection of data patterns and the prediction of measurement outcomes [67]. Machine learning algorithms have been developed to extract information from raw EEG data to identify various brain states and assist in the diagnosis of several conditions [41]. After extracting key features, machine learning algorithms can distinguish between normal and MCI-related EEG rhythms. Support vector machines (SVM), random forests (RF), and K-nearest neighbor (KNN) are three common machine learning algorithms that can be used to generate classifiers from labeled training data [68]. These algorithms are able to determine whether new EEG data are related to MCI based on previous examples. By analyzing temporal and spatial connections, these systems can detect early or minor signs of MCI in EEG data that humans may not notice. Its personalized approach permits early intervention and customized treatment strategies. Some of the reliable machine-learning models that have been utilized for classifying, diagnosing, and tracking the progression of AD based on EEG data are briefly discussed [42].

Convolutional neural networks (CNN) have been shown to be effective in classifying EEG signals for various neurological disorders, including AD [44],[45]. This model can learn complex patterns from raw EEG signals and achieve high classification accuracy. A recent study by Shan et al. [46] proposed a novel dynamical spatio-temporal graph, CNN, to fully exploit the constrained spatial topology of functional connectivity and the discriminative dynamic temporal information represented by 1D convolution. The model outperformed cutting-edge approaches in terms of classification performance by 92.3%. This model not only helps detect AD but also helps better understand the impact of natural aging on brain network features. It achieved better classification performance than state-of-the-art methods [46].

The deep neural network (DNN)-based model is another model that uses EEG signals to detect the early stage [59] and to predict the progression of AD [60]. The EEG signals of the subjects were fed into the model, which then categorized them into two groups: MCI and healthy controls (HC). In comparison to a shallow neural network, the proposed DNN approach was evaluated by the authors and demonstrated superior performance [61]. Developing a suitable training approach for DNNs has been a significant hurdle despite some progress in the field [47],[48].

The first pre-training method introduced to address this issue is the Deep Belief Network (DBN). A DBN is composed of multiple stacked restricted Boltzmann machines (RBM) that are trained in an unsupervised manner. The network learns to reconstruct inputs probabilistically using the features obtained at each layer. In addition to discovering generative features, DBNs can be utilized for discriminative prediction tasks [42],[50].

A study by Alessandrini et al. [51] demonstrated the effectiveness of using a recurrent neural network (RNN) in handling significantly corrupted data when it is pre-filtered using a robust principal component analysis (RPCA) algorithm. The RPCA was chosen because of its ability to eliminate outliers from the signal. Initially, an RNN model that worked on EEG data was preprocessed using traditional PCA. Subsequently, using corrupted data as input, the outlier components are filtered out using RPCA. The results revealed that even with up to 20% data corruption, RPCA was able to enhance detection accuracy by approximately 5% compared to the baseline PCA [51].

### 4.5. Limitations

While this study provides valuable insights into the research trends and themes related to EEG and MCI, there are a few limitations. First, only papers written in English and listed in the Scopus databases during the study period were included in the bibliometric analysis. This implies that not all pertinent research may have been included, especially those written in non-indexed journals or not available in English. Second, bibliometric analysis depends on metadata that is accurate and consistent across databases and publications, such as author names and publication dates. This may cause inconsistencies and mistakes during the analysis. Third, the research themes in this study were derived from keyword co-occurrence thematic analysis without deeper analysis. Finally, bibliometric analysis does not provide insights into the content or context of the studies, which may be important for scoping reviews that aim to identify research gaps and inform future research directions. To address this limitation, we employed the PAGER framework to ensure a more comprehensive scoping review of the research on EEG and MCI.

## 5. Conclusions

With the aid of bibliometric analysis, we carried out a scoping review (ScoRBA) and used the PAGER framework to present our findings. We concluded that EEG-based brain mapping is a useful technique and is essential for advancing the knowledge, diagnosis, and treatment of AD, including in individuals with MCI. Cognitive processes and pathology related to AD and MCI can be better understood using EEG techniques, such as ERP and QEEG. Machine learning techniques used on EEG data have the potential to identify and forecast MCI and AD.
